# The role of complement in the immunopathogenesis of acetylcholine receptor antibody-positive generalized myasthenia gravis: bystander or key player?

**DOI:** 10.3389/fimmu.2025.1526317

**Published:** 2025-04-15

**Authors:** Iliana Michailidou, Anastasia Patsiarika, Evangelia Kesidou, Marina Kleopatra Boziki, Dimitrios Parisis, Christos Bakirtzis, Elisabeth Chroni, Nikolaos Grigoriadis

**Affiliations:** ^1^ Laboratory of Experimental Neurology and Neuroimmunology, AHEPA University Hospital, Aristotle University of Thessaloniki, Thessaloniki, Greece; ^2^ AstraZeneca, Medical Affairs Department Rare Disease, Athens, Greece; ^3^2^nd^ Department of Neurology, AHEPA University Hospital, Aristotle University of Thessaloniki, Thessaloniki, Greece; ^4^ Department of Neurology, Medical School, University of Patras, Patra, Greece

**Keywords:** myasthenia gravis, complement system, anti-acetylcholine receptor antibodies, membrane attack complex, therapy

## Abstract

The complement system is a key component of the innate immune system. In antiacetylcholine receptor (AChR) antibody-positive (Ab+) generalized myasthenia gravis (MG), complement activation has long been considered a principal driver of pathology. Understanding the role of complement in AChR-Ab+ generalized MG has gained increasing importance in recent years, as anticomplement drugs have been approved for clinical use or are undergoing phase II/III clinical trials. This review aims to discuss recent and previous findings on the role of complement in AChR-Ab+ MG pathology, including its interaction with pathogenic antibodies and mechanisms beyond the classical pathway activation.

## Introduction

Myasthenia gravis (MG) is a rare, chronic neuromuscular autoimmune disease with a median global prevalence of 10 per 100,000 and an in-hospital mortality rate of 1.8%. The hallmark of MG pathology is defective transmission at the neuromuscular junction. MG is characterized by fatigability and fluctuating weakness of the ocular, bulbar, respiratory, and limb muscles. Most people with MG (pwMG) develop ocular symptoms at some point during the disease course. Approximately 80% of pwMG with ocular onset progress to generalized MG within 2 years of disease onset (1–3).

MG is a T-cell-dependent disease in which CD4+ T cells produce cytokines and promote B-cell differentiation into plasma cells (4). The trigger for T-cell activation and MG development remains unknown but has been linked to thymomas (1, 5). Plasma cells produce autoantibodies that target neuromuscular junction proteins, a specialized synapse that transmits nerve impulses to muscles across the synaptic cleft. MG autoantibodies and serum molecules, including complement proteins, can bind to and become activated at the neuromuscular junction, as this structure lacks protection from the blood–nerve barrier (4).

MG autoantibodies vary in IgG subclasses, protein targets, and pathogenic actions, forming the basis for disease subgroup classification. In particular, autoantibodies against the acetylcholine receptor (AChR) belong to the IgG1 and IgG3 subclasses (6) and account for 85% of cases. There are three pathogenic mechanisms by which AChR autoantibodies lead to MG: antibody blocking, antigenic modulation, and complement activation as described below. Another 5% of all pwMG have autoantibodies against muscle-specific kinase (MuSK), which belongs to the IgG4 subclass (7). IgG4 antibodies have a limited capacity to interact with complement proteins and are therefore considered noncomplement-fixing antibodies (8). Additionally, 1%–5% of pwMG have autoantibodies targeting low-density lipoprotein receptor-related protein 4 (LRP4), which belongs to the IgG1 subclass (9). Approximately, 10% of pwMG are diagnosed without detectable autoantibodies and are classified as seronegative.

The complement system is a key effector mechanism in anti-AChR antibody-positive (Ab+) MG (4, 10). Complement activation may occur as an early and crucial event in MG (11–13), while the impact of autoantibodies alone remains poorly understood (14). Therefore, a causal rather than a bystander role for complement in MG pathology should be considered, given its involvement in primarily degenerative processes such as autophagy and synaptic pruning (15, 16). This review discusses the mechanisms of complement-mediated damage at the neuromuscular junction in the presence or absence of autoantibodies.

## AChR autoantibody-mediated pathogenic mechanisms in MG

AChRs are heteropentamers consisting of two α-subunits and one β-, δ-, and either γ-subunit (in embryonic muscle) or ϵ-subunit (in adult muscle), organized around a central ion channel. These receptors are located at the postsynaptic membrane of the neuromuscular junction and facilitate the generation of a muscle fiber action potential upon binding of Ach, which is released by the firing nerve ending. In MG, the anti-AChR antibody response is polyclonal; however, at least half of the AChR autoantibodies target the α-subunits, specifically the N-terminal region of AChR-α, which includes the main immunogenic region (MIR). The MIR is a cluster of conformation-dependent epitopes recognized by functionally and structurally heterogenous monoclonal anti-MIR antibodies (17). These anti-MIR antibodies may promote AChR clustering at the neuromuscular junction by binding to the α-subunits of adjacent receptors (18).

MG pathology may result from the direct blockade of AChRs by autoantibodies, which inhibits ACh binding to its receptor. Steric interference with ACh-AChR binding may lead to an activity-dependent reduction in neuromuscular junction efficacy, preventing striated muscle contraction and ultimately causing denervation of the affected muscle (19, 20). Anti-AChR autoantibodies have also been found to slow AChR channel closure, leading to abnormal AChR desensitization and the formation of miniature rather than normal endplate currents (21). In MG, miniature endplate currents are insufficient to reach the threshold level necessary for generating a muscle fiber action potential (22).

However, most MG autoantibodies do not block the binding of ACh to its binding sites. This may—at least in part—explain the lack of a significant relationship between antibody titers and disease severity (23, 24). PwMG in remission may present with the highest antibody abundance (25), whereas severe disease is primarily associated with the loss of AChRs and the destruction of junctional folds (26). Previous studies have categorized pathogenic antibodies as blocking or binding using standard immunoprecipitation assays to quantify binding antibodies and assays to measure antibodies that block α-bungarotoxin binding to receptors (24, 25, 27). Although the degree of AChR blockade was significantly correlated with the generalization of muscle weakness (25), people with severe generalized MG had either both blocking and binding antibodies or binding antibodies alone (28). Whether blocking and binding MG antibodies bind to distinct AChR sites with higher or lower agonist affinity—and whether the two types of antibodies act synergistically to exert a functional action on AChR—remains to be determined.

The second hypothesis for MG pathogenesis is based on the ability of anti-AChR autoantibodies and their divalent F(Ab′)2 fragments to modulate—rather than block—AChRs (29). Antigenic modulation activity in MG has been linked to antibody titers (30). Autoantibodies targeting AChRs are believed to accelerate the natural AChR degradation cycle by binding to the MIR and inducing AChR cross-linking (18). AChR cross-linking is followed by internalization and lysosomal degradation of the autoantibody-bound receptors, which—similar to the first hypothesis—reduces AChR densities at the neuromuscular junction (29). Notably, studies in rodents have indicated that increased AChR internalization is compensated by enhanced AChR synthesis, resulting in similar AChR levels before and after internalization (31, 32). However, other studies have reported contradictory data (33, 34). A key question remains: what is the impact of coexisting pathological mechanisms on the synthesis and insertion of newly synthesized AChRs at the neuromuscular junction? In addition, further research is needed on the kinetics and recycling potential of internalized AChRs to better understand the exact contribution of the modulatory effect of autoantibodies on MG pathogenesis and progression.

## Complement-mediated pathogenic mechanisms in MG

The complement system consists of more than 30 proteins found in plasma and on cell surfaces, which enhance the activity of antibodies and phagocytic cells in removing microbes, immunocomplexes, and dead or modified self-cells. It primarily serves the innate immune system while also interacting with components of the adaptive immune system to regulate antibody-mediated responses. Complement proteins, mainly produced by hepatocytes, have both effector and regulatory roles (35).

**Figure 1:**
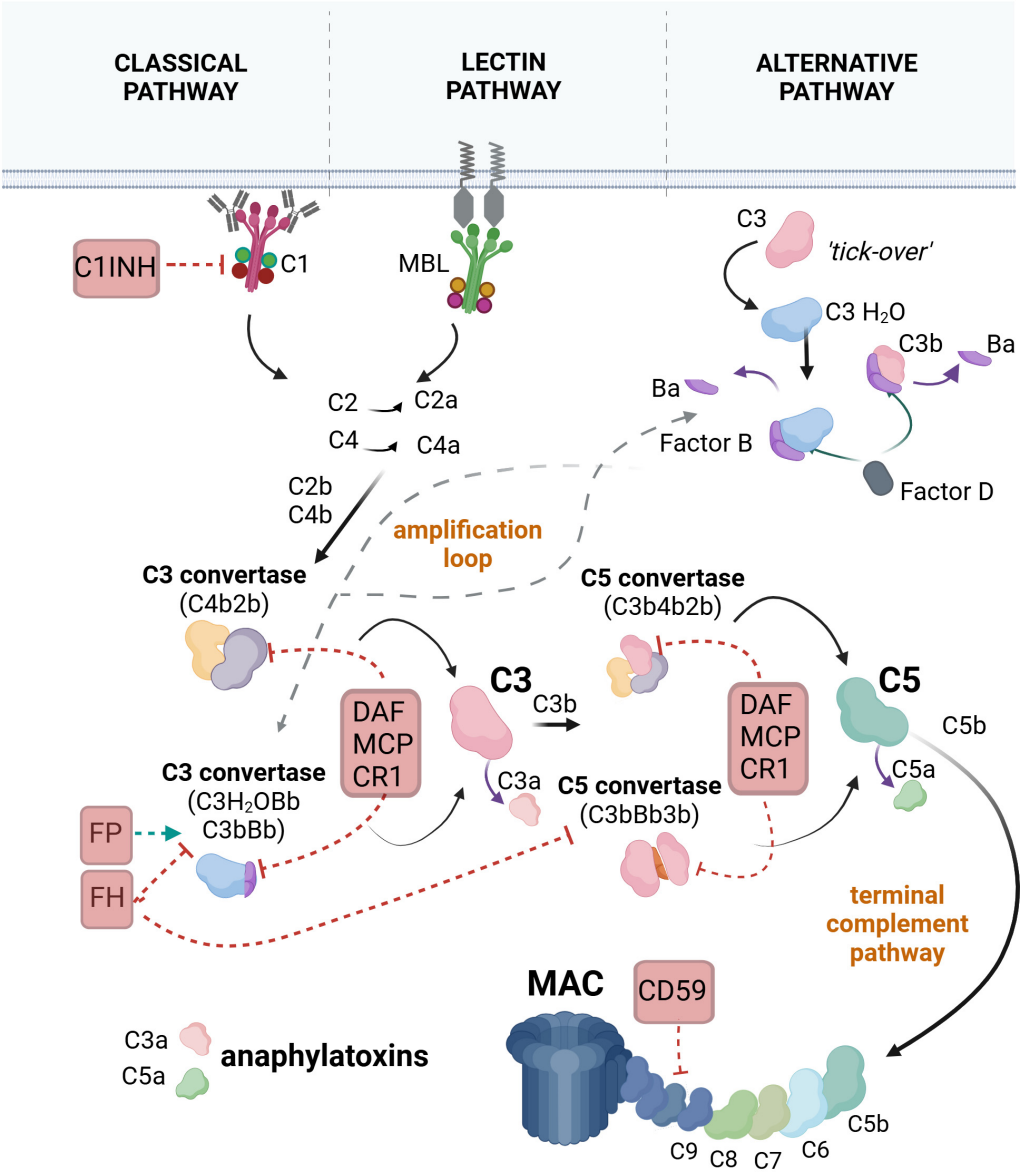
The pathways of complement system activation. In the classical pathway, C1q, a subunit of the C1 complex, binds to an antibody-fixed cell. This binding activates the proteases C1r and C1s (also components of the C1 complex). C1s cleave the C4 protein into C4a and C4b. C4b binds covalently to the target and attracts C2, which is cleaved by C1s into C2a and C2b. C2b binds to C4b to form the C4b2b complex, also known as C3 convertase. C3 convertase cleaves C3 into C3a and C3b. C3b may bind to the C3 convertase, forming the C4b2b3b complex, also known as C5 convertase. C5 convertase cleaves C5 into C5a and C5b. C5b associates with C6, C7, C8, and C9 to form the final product of complement activation, the membrane attack complex (MAC). In the lectin pathway, mannose-binding lectin (MBL) or ficolins bind to carbohydrate patterns on the target cell, leading to the activation of MBL-associated serine proteases (MASP)1 and MASP2, which are complexed with MBL. MASPs cleave C4 and C2, forming C3 convertase. The pathway continues with the generation of the C5 convertase and the formation of MAC. In the alternative pathway, inactive C3 undergoes spontaneous hydrolysis, forming C3(H_2_O). Factor B binds to C3(H_2_O), leading to its cleavage by factor D into Ba and Bb, resulting in the formation of the fluid-phase C3 convertase, C3(H_2_O)Bb. C3(H_2_O)Bb converts C3 into C3a and C3b, with some C3b molecules binding to the target and associating with factor B, which is subsequently cleaved by factor D to form the C3 convertase C3bBb. The pathway progresses with the formation of the C5 convertase (C3bBb3b), cleavage of C5, and the assembly of the MAC. Selected regulators of the complement cascade that interfere with different steps of the cascade are illustrated. C1 inhibitor (C1INH) promotes the decomposition of the C1 complex. Decay-accelerating factor (DAF), membrane cofactor protein (MCP), and complement receptor 1 (CR1) accelerate the decay of the C3 and C5 convertases. CD59 inhibits the formation of MAC. Factor H (FH) prevents the assembly of the alternative pathway C3 and C5 convertases, while factor P (properdin) stabilizes the alternative pathway C3 convertase, thereby promoting the amplification loop. Created with BioRender.com.

Activation of complement occurs through three pathways: the classical, lectin, and alternative pathways (**Figure 1**). The classical pathway is triggered when C1q binds to immune complexes or “eat me” signals displayed by apoptotic cells. Similarly, the lectin pathway is initiated when mannose-binding lectin (MBL) or ficolins bind to carbohydrate patterns on the cell membrane. The alternative pathway is activated by the slow spontaneous hydrolysis (tick-over) of C3 to C3(H_2_O), allowing a continuous but subtle complement activation that contributes to immune surveillance and can amplify neuroinflammatory responses. All complement pathways ultimately lead to three key outcomes: opsonization of a target for phagocytosis via the covalently bound C3b component, induction or amplification of inflammation through the released C3a and C5a anaphylatoxins, and target cell damage via the membrane attack complex (MAC). MAC is a pore-forming structure that integrates into the lipid bilayer of the plasma membrane, causing lysis (13, 35) or triggering proinflammatory responses when formed at sublytic levels (36).

Complement can have the potential to harm self-tissues; therefore, its activation is tightly regulated (**Figure 1**) to eliminate pathogens or “unwanted” cells without injuring the host. When this balance is disrupted, excessive complement activation can lead to tissue injury and contribute to the pathology of various diseases (35).

**Figure 2:**
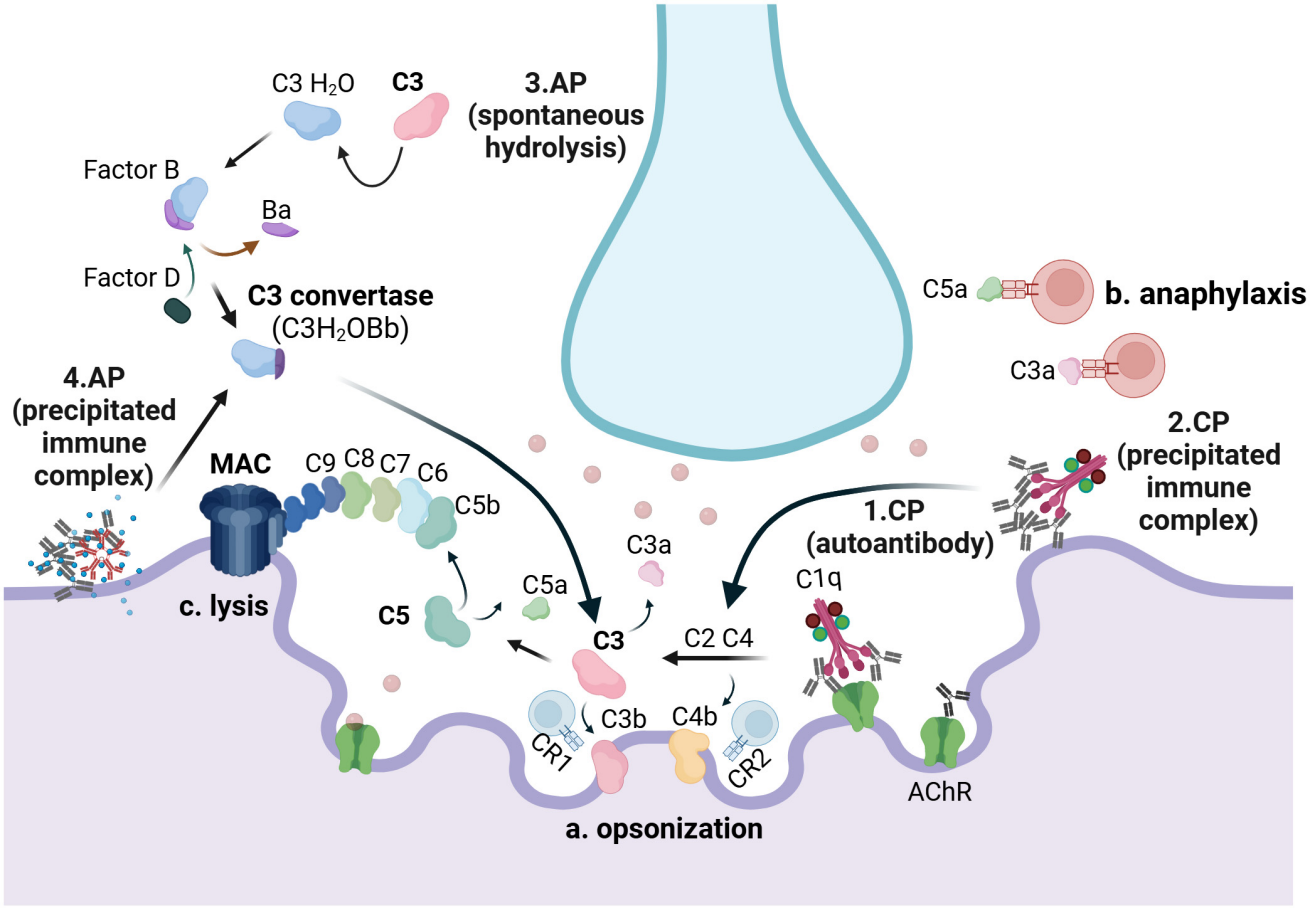
The complement system in AChR-Ab+ generalized myasthenia gravis: C1q binds to autoantibodies attached to AChRs (1) and to precipitated immune complexes (2), when present, at the neuromuscular junction, thereby activating the classical pathway (CP). Hydrolysis of C3, either spontaneously (3) or triggered by precipitated immunocomplexes (4), induces activation of the alternative pathway (AP). CP activation results in the opsonization of the muscle membrane by C4b and C3b, which serve as potent activators of CR1- and CR2-bearing lymphocytes **(a)**. Both the CP and the AP result in the release of C3a, a product of C3 cleavage, and C5a, a product of C5 cleavage, which may interact with immune cells bearing C3a and C5a receptors, respectively **(b)**. Additionally, both pathways contribute to the formation of the membrane attack complex (MAC), composed of C5b, C6, C7, C8, and C9, which serves as the key driver of neuromuscular junction damage **(c)**. CP, classical pathway; AP, alternative pathway. Created with BioRender.com.

Numerous studies assessing the levels of complement activation products and regulators in plasma (37–41) and biopsied material (42–46) from individuals with AChR-Ab+ MG have confirmed that the activated complement system is a key mediator of AChR-Ab+ MG pathology (10, 13, 47). Its physiological role in eliminating antibody-targeted pathogens (35) leads to binding and activation at the autoantibody-fixed AChR-Ab+ MG neuromuscular junction, resulting in tissue damage (42–44). Activation occurs via the classical pathway when the C1q molecule binds to the Fc region of IgG1 and IgG3 autoantibodies attached to AChRs (48). Since optimal C1q binding requires multiple IgG molecules to bind one C1q, the clustering of autoantibody-bound AChRs on the postsynaptic membrane plays an important role in classical pathway activation (49). Activation of the classical pathway leads to the assembly of the pore-forming MAC, which serves as the key effector of complement-mediated damage in AChR-Ab+ MG. MAC induces direct destruction of the postsynaptic membrane, reducing AChRs and voltage-gated channels, and significantly contributing to the widening of the postsynaptic cleft and muscle denervation (50). Next to the role of MAC, activation of the classical pathway results in the covalent binding of C4b and C3b opsonins on the muscle surface. These opsonins be recognized by complement receptor type 1 (CR1)- and type 2 (CR2)-bearing cells, including T and B cells. Furthermore, activation leads to the release of C3a and C5a, which modulate leucocyte responses by binding to C3aR and C5aR (**Figure 2**). It remains unclear whether, in AChR-Ab+ MG, C1q activates complement in the absence of antibodies by binding to motifs or molecules exposed on muscle endplates. This possibility might explain its deposition on the motor endplates in the intercostal muscle of amyotrophic lateral sclerosis (ALS) donors (51). Alternatively, C1q activation may be linked to activity patterns at the neuromuscular junction, a mechanism previously suggested to underlie its role in the synapse elimination in the central nervous system by microglia (52).

The molecular contribution of MAC to MG pathology has been inferred from studies conducted in experimental autoimmune myasthenia gravis (EAMG) models (53, 54). Pharmacological inhibition of MAC in EAMG has been achieved using C5 inhibitors, such as the 4G2 monoclonal antibody (55) and the rEV576 compound (56), both of which block rat C5 and thereby inhibit MAC formation as well as C5a-induced anaphylaxis. Additionally, MAC inhibitors, including the 7E5 monoclonal antibody targeting human C6 (57) and the TPP1820 monoclonal antibody targeting mouse C7 (58), have also been used to suppress MAC activity. The administration of C5, C6, or C7 terminal complement pathway inhibitors in rodents mitigated EAMG.

Moreover, inducing EAMG in mice deficient in C5 (59) or C6 (53), both components of the terminal pathway, resulted in a reduced incidence of severe disease and better preservation of AChRs compared with wildtype littermates. In contrast, animals lacking the *CD59a* gene, which encodes the CD59 regulator of MAC formation, exhibited high levels of C9 immunoreactivity at the diaphragm neuromuscular junctions, indicating MAC deposition (60). In line with these data, C3 or C4 knockout mice, which are unable to form MAC, exhibited autoantibody deposition at the neuromuscular junctions when immunized with anti-AChR antibodies but remained resistant to EAMG development, with no alterations in AChR densities (61). This demonstrates the role of complement in the induction and progression of EAMG. In contrast, mice deficient in the CD55/decay-accelerating factor (DAF), a regulator of C3 and C5 convertase decay, exhibited excessive MAC formation and extensive damage to the postsynaptic membrane (60). Notably, EAMG rodents deficient in the C3 protein exhibited reduced serum IgG levels, including anti-AChR IgGs, and a decreased number of B cells, impacting the adaptive immune system (61). Collectively, these findings indicate that complement serves as the primary effector mechanism in EAMG (**Table 1**).

**Table 1:** Published data support an AChR-Ab-independent role of complement in generalized MG and suggest a causal role in EAMG pathology.

Myasthenia gravis	
SNPs rs344555 and rs3745568 in the C3 gene were associated with a higher risk of MG.	Yue et al. (100)
Complement attack on both epithelial and myoid cells precedes MG autoantibody diversification.	Leite et al. (101)
Complement proteins are deposited on the motor endplates of seronegative MG donors.	Nagaoka et al. (12); Hoffmann et al. (74)
C9 deposits at an endplate region are inversely related to the structural integrity of the junctional folds in MG.	Sahashi et al. (45)
Complement and immune complexes are present on the endplates of seronegative MG people.	Tsujihata et al. (43)
Immunohistochemical analysis of endplates of pwMG showed the presence of MAC at all endplates.	Nakano and Engel (102)
Experimental models of myasthenia gravis	
C3 and C4 KO mice showed IgGs on the neuromuscular junction but were unable to develop EAMG following AChR immunization; anti-AChR Ab production was suppressed in C3KO mice.	Tuzun et al. (61)
C5 deficiency prevents EAMG induced by AChR immunization.	Christadoss (59)
C6 deficiency prevents passive EAMG induction.	Chamberlain-Banoub et al. (53)
Deficiency for Daf1 and/or Cd59a determines the severity of passively induced EAMG.	Morgan et al. (54); Kusner et al. (103)
Treatment of active or passive EAMG with a C5 inhibitor reduced the amounts of C9 deposits at the neuromuscular junction but did not alter total serum AChR Ab levels.	Soltys et al. (56)
Other autoantibody-independent neuromuscular diseases and related models	
Amyotrophic lateral disease (ALS): MAC deposits were detected on innervated motor endplates in the intercostal muscles of ALS donors.	Bahia el Idrissi et al. (51)
ALS: Y402H variant of CFH was associated with a higher risk of ALS.	Baird et al. (104)
Experimental model of ALS: activated C3 and C1q deposited on the neuromuscular junction of a mouse model for familial ALS, at the presymptomatic stage.	Heurich et al. (105)
Experimental models of Charcot Marie Tooth (CMT): C1q, C3, and C9 deposits detected on sciatic nerves of transgenic mouse models of CMT1A	Michailidou et al. (106)

## Limiting complement activation vs. lowering AChR autoantibody titers for suppression of MG

Since 2017, terminal complement pathway inhibition has been a therapeutic option for individuals with AChR-Ab+ MG. Eculizumab, a C5 inhibitor, was the first anticomplement drug approved for the treatment of refractory generalized MG in people aged 6 years and older with AChR-Ab+. Four mutations on eculizumab led to the development of ravulizumab, the first long-acting C5 inhibitor, which received regulatory approval for AChR-Ab+ generalized MG in 2022. Ravulizumab is administered via intravenous infusion every 8 weeks, offering a longer dosing interval compared to eculizumab, which is given every 2 weeks (62, 63). In 2023, a year later, zilucoplan, a peptidic C5 inhibitor, was authorized for daily self-administration via subcutaneous injection. Zilucoplan has a favorable safety profile and has been shown to maintain or improve MG symptoms in pwMG switching from intravenous C5 inhibitor therapy, based on data from the interim analysis of a phase C3b study (64). The results of phase III trials (65, 66), along with subsequent market authorizations of the aforementioned C5 inhibitors, the ongoing preclinical testing of the CP010 C6 inhibitor (https://gravitoncorp.com), and the extensive list of complement inhibitors that have been evaluated in clinical phase I/II and III trials or are currently undergoing phase II/III clinical testing—including ALXN1720 (NCT05556096), iptacopan (NCT06517758), DNTH103 (NCT06282159), and forelimb/cemdisiran (NCT05070858) inhibitors—suggest that anticomplement therapeutics may play a central role in the treatment of AChR-Ab+ generalized MG. Several phase III trials were followed by corresponding open-label extension studies (**Table 2**).

**Table 2:** Clinical trials of complement inhibitors for the treatment of generalized MG.

NCT number	Study title	Study status	Interventions	Target	Phase	Study type	Primary endpoint results	Completion date
**NCT06607627**	PK, PD, safety, and efficacy study of gefurulimab in pediatric patients with AChR+ generalized myasthenia gravis	Recruiting	Gefurulimab	C5	Phase 3	INT	N/A	12 June 2029
**NCT06517758**	A phase III study to investigate efficacy, safety, and tolerability of iptacopan compared with placebo in participants aged 18 to 75 years with gMG	Recruiting	Iptacopan	Factor B	Phase 3	INT	N/A	12 January 2029
**NCT05070858**	A study to test how safe pozelimab and cemdisiran combination therapy and cemdisiran alone are and how well they work in adult patients with generalized myasthenia gravis	Recruiting	Pozelimab + cemdisiran| cemdisiran| pozelimab	C5	Phase 3	INT	N/A	23 March 2028
**NCT06282159**	A phase 2 study to evaluate DNTH103 in adults with generalized myasthenia gravis (MAGIC)	Recruiting	DNTH103	C1s	Phase 2	INT	N/A	December 2027
**NCT05556096**	Safety and efficacy of ALXN1720 in adults with generalized myasthenia gravis	Active not recruiting	Gefurulimab	C5	Phase 3	INT	N/A	11 August 2027
**NCT05218096**	Study of ALXN2050 in adult participants with generalized myasthenia gravis	Terminated	Vemircopan	Factor D	Phase 2	INT	N/A	03 April 2024
**NCT06435312**	An open-label extension study to evaluate subcutaneous zilucoplan in pediatric participants with generalized myasthenia gravis	Enrolling by invitation	Zilucoplan	C5	Phase 3	INT	N/A	07 December 2027
**NCT06055959**	A study to evaluate subcutaneous zilucoplan in pediatric participants with generalized myasthenia gravis	Recruiting	Zilucoplan	C5	Phase 2|phase 3	INT	N/A	25 December 2026
**NCT04225871**	Open-label extension of zilucoplan in subjects with generalized myasthenia gravis	Active not recruiting	Zilucoplan	C5	Phase 3	INT	A total of 188 (94%) patients experienced a TEAE. The most common TEAEs were: MG worsening (*n* = 52, 26%) and COVID-19 (*n* = 49, 25%) (77)	02 June 2026
**NCT06471361**	A study to evaluate the safe and effective use of a zilucoplan auto-injector by study participants with generalized myasthenia gravis	Completed	Zilucoplan	C5	Phase 3	INT	N/A	03 February 2025
**NCT05514873**	An open-label study to evaluate the safety, tolerability, and efficacy of subcutaneous zilucoplan in participants with generalized myasthenia gravis who were previously receiving intravenous complement component 5 inhibitors	Completed	Zilucoplan	C5	Phase 3	INT	Based on an interim analysis, four (50.0%) participants reported treatment-emergent adverse events; one treatment-emergent adverse event was considered treatment-related (injection-site pruritus) (64)	23 October 2024
**NCT04115293**	Safety, tolerability, and efficacy of zilucoplan in subjects with generalized myasthenia gravis	Completed	Zilucoplan	C5	Phase 3	INT	Least squares mean (95% CI) change from baseline to week 12 in MG-ADL total score—placebo: − 2.30 (− 3.17 to − 1.43); zilucoplan: − 4.39 − 5.28 to −3.50) (66)	30 December 2021
**NCT03315130**	Safety and efficacy study of RA101495 in subjects with generalized myasthenia gravis	Completed	Zilucoplan	C5	Phase 2	INT	Mean (standard deviation) change from baseline in QMG total score at week 12—placebo: − 3.2 (1.2); zilucoplan at 0.1 mg/kg: − 5.5 (1.2); zilucoplan at 0.3 mg/kg: − 6.0 (1.2) (107)	19 November 2020
**NCT06312644**	Study of Ultomiris^®^ (ravulizumab) safety in pregnancy	Recruiting	Ravulizumab	C5	N/A	OBS	N/A	24 October 2034
**NCT04202341**	Registry of participants with generalized myasthenia gravis treated with alexion C5 inhibition therapies (C5ITs)	Recruiting	Eculizumab/ravulizumab	C5	N/A	OBS	N/A	31 December 2029
**NCT05644561**	Evaluation of pharmacokinetics, pharmacodynamics, efficacy, safety, and immunogenicity of ravulizumab administered intravenously in pediatric participants with generalized myasthenia gravis (gMG)	Recruiting	Ravulizumab	C5	Phase 3	INT	N/A	31 July 2028
**NCT03920293**	Safety and efficacy study of ravulizumab in adults with generalized myasthenia gravis	Completed	Ravulizumab	C5	Phase 3	INT	Least squares mean (95% CI) change from baseline in MG-ADL total score at week 26:—placebo: − 1.4 (0.37); ravulizumab: − 3.1 (0.38) (65)	25 May 2023
					OLE	INT	Based on an interim analysis, the least squares mean (95% CI) change from the OLE baseline in MG-ADL total score to week 60—ravulizumab/ravulizumab: − 4.0 (− 4.8, −3.1); placebo/ravulizumab: − 1.7 (− 2.7, − 0.8) (108)	
					OLE	INT	Least squares mean (95% CI) change from OLE baseline in MG-ADL at week 164—ravulizumab/ravulizumab − 4.0 (− 5.3, − 2.8); placebo/ravulizumab − 2.1 (− 3.3, − 0.9) (109)	
**NCT03759366**	A phase 3 open-label study of eculizumab in pediatric participants with refractory generalized myasthenia gravis (gMG)	Completed	Eculizumab	C5	Phase 3	INT	Mean (standard deviation) change from baseline in QMG total score at week 26 regardless of rescue treatment—eculizumab: − 6.1 (4.56) (110)	06 November 2023
**NCT02301624**	Extension study of ECU-MG-301 to evaluate safety and efficacy of eculizumab in refractory generalized myasthenia gravis	Completed	Eculizumab	C5	Phase 3	INT	Count of participants with treatment-emergent adverse events (percentage)—placebo/eculizumab: 59 (96.7%); eculizumab/eculizumab: 55 (98.2%) (111)	15 January 2019
**NCT01997229**	Safety and efficacy of eculizumab in refractory generalized myasthenia gravis (REGAIN study)	Completed	Eculizumab	C5	Phase 3	INT	Least squares mean (standard error) change from baseline in MG-ADL total score at week 26 by worst rank analysis of covariance (ANCOVA)—placebo: 68.3 (4.49); eculizumab: 56.6 (4.53) (112)	June 2016

Only clinical trials registered in ClinicalTrial.gov were included. Phase 2 clinical trials that were followed by a corresponding phase 3 trial were not included.

INT, interventional; OBS, observational; OLE, open-label extension; QMG, quantitative myasthenia gravis; MG-ADL, myasthenia gravis activities of daily living; N/A, not applicable; CI, confidence interval. NCT number, National Clinical Trial Identifier Number.

Terminal complement pathway inhibitors are promising for AChR-Ab+ MG therapy because they block the destructive MAC while preserving upstream complement mechanisms. This ensures physiological protection against infection and may facilitate immune complex clearance in MG. Activated C3 is known to mediate immune complex clearance via CR1-receptor-bearing cells (67) and aids in their solubilization to prevent precipitation. The solubilization of preformed immune complexes is considered a primary function of the alternative complement pathway (68), though the classical pathway is also involved (69). Precipitated immune complexes, particularly those formed by IgG1 or IgG3 antibodies, can have significant pathological impacts on various tissues (70), as they activate the complement system. Immune complexes and C3 have previously been identified in forelimb muscles in EAMG (45) and within degenerating junctional folds in humans (71, 72). Additionally, IgG, C3, and C9 were detected at the limb muscle endplates of pwMG (43). Interestingly, some pwMG with immune complexes and activated complement exhibited no detectable serum anti-AChR antibodies (43), suggesting that insufficient immune complex clearance, rather than autoantibody production, may contribute to complement-mediated damage in seronegative pwMG (**Table 1**). However, the possibility cannot be excluded that complement activation at the neuromuscular junction of seronegative pwMG is triggered by an unknown autoantibody targeting the synapse or that the sensitivity of current antibody quantification assays is insufficient to detect low levels of circulating antibodies. Live cell-based assays for detecting anti-AChR antibodies may serve as a valuable tool for diagnosing patients with radioimmunoassay-double seronegative MG (73). Furthermore, the use of intercostal muscle biopsy to identify complement depositions at the neuromuscular junction (74) may need to be considered for diagnosis and/or therapeutic decision-making in patients for whom live cell-based assay has also failed to detect antibodies.

The advantage of selectively blocking only the damaging arm of the complement system, rather than promoting broad immunosuppression, has been inferred by real-world studies demonstrating sustained improvement (65, 75–77) and a reduced need for corticosteroids (78–85) in people with AChR-Ab+ generalized MG receiving C5 inhibitor therapies. In contrast, plasmapheresis, which depletes MG autoantibodies from the serum, is essential for symptom relief during a crisis but provides only a short-lasting (approximately 6 weeks) effect, as symptoms return once autoantibody levels revert to baseline (86). Similarly, intravenous immunoglobulin (IVIg) and other immunosuppressive therapies—such as corticosteroids, azathioprine, and the neonatal Fc receptor (FcRn) inhibitors—regulate autoantibody levels and indirectly modulate complement-dependent damage. However, these drugs cannot block the effects of residual autoantibodies bound to the neuromuscular junction or suppress potential activation of the antibody-independent alternative pathway, meaning they do not specifically block complement-mediated effects (11). Notably, these treatments are ineffective in reducing alternative pathway activation, which implies the effects of initial classical pathway activation. This has significant implications for MG, particularly given data demonstrating changes in serum levels of the alternative pathway regulator properdin in pwMG (87). Remarkably, robust classical pathway activation may bypass the effect of the alternative pathway (88), suggesting that alternative complement pathway inhibitors may be beneficial only when classical pathway activation is modest. This may explain the lack of efficacy of vemircopan, a factor D inhibitor, in a phase II clinical trial. A phase III clinical trial evaluating the effects of iptacopan, a factor B inhibitor, is currently underway, and its results are awaited. The anti-B-cell rituximab is particularly effective in MuSK-Ab+ MG and is often prescribed for individuals with AChR-Ab+ refractory MG based on long-term clinical experience (89, 90). However, rituximab may promote complement activation as an adverse reaction, which could explain the lack of response in some pwMG (89), including those with refractory MG who are double seronegative for anti-AChR and anti-MuSK antibodies (91). It is noteworthy that anti-LRP4 antibodies can—at least partially—activate the complement system (92), but the impact of complement-mediated mechanisms in anti-LRP4 Ab+ MG remains underrecognized in clinical practice.

Targeting the terminal complement pathway in AChR-Ab+ MG may regulate the disease course beyond MAC formation. Zilucoplan has been shown to prevent C5b6 formation through plasmin-mediated noncanonical C5 activation, thereby interfering with red blood cell hemolysis (93). Meanwhile, eculizumab has been found to induce functional pathways related to antioxidant activity, cholesterol transfer, and cellular detoxification, while downregulating pathways associated with antigen binding. In addition, eculizumab reduced leukotriene production, possibly due to the inhibition of C5a-induced anaphylaxis (94).

Studies indicate that despite current therapeutic interventions for AChR-Ab+ generalized MG, a proportion of people, approximately 10% or higher, do not respond adequately to treatment. These pwMG may remain refractory, develop drug dependency, or experience intolerable side effects (95–98). For those receiving C5 inhibitors, vaccination against all meningococcal strains should be administered as close as possible to therapy initiation. When the interval between vaccination and treatment is insufficient, prophylactic antibiotics are strongly recommended (99). This is because activation of upstream complement functions alone is insufficient for protection against *Neisseria* species, a group of bacteria that requires activation of the terminal pathway and can cause meningococcal sepsis or meningitis. Since no reliable biomarkers of MG pathology are currently available, the present treatment approach relies on trial and error. There is an urgent need for personalized MG treatment, which may be achieved by gaining deeper insights into both the beneficial and adverse effects of existing therapies.

Overall, terminal complement activation is destructive, making its inhibition a primary goal in treating people with AChR-Ab+ generalized MG. Identifying biomarkers to guide therapeutic decisions and understanding inhibitor-induced molecular actions may help improve treatment responses.

## Concluding remarks

Complement plays a crucial role in MG pathology, as the formation of MAC at MG endplates can lead to the destruction of the neuromuscular junction. AChR blocking and antigenic modulation trigger complement activation in MG. In addition to the classical pathway, the role of the activated alternative pathway in MG pathology requires further evaluation, as antibody-targeting therapies may not efficiently suppress it.
